# Electrochemotherapy of spinal metastasis using transpedicular approach: a preclinical safety animal study

**DOI:** 10.1186/s41747-025-00607-9

**Published:** 2025-09-04

**Authors:** Frederic Deschamps, Enzo Gautreau, Lambros Tselikas, Baptiste Bonnet, Paul Beunon, Adlane Feddal, Thierry de Baere, Amelie Gaudin, Lluis M. Mir

**Affiliations:** 1 Gustave Roussy, Interventional Radiology Department, Paris-Saclay University, 114 rue Edouard Vaillant, 94805 Villejuif, France; 2 Gustave Roussy, UMR 9018—Metabolic and Systemic Aspects of Oncogenesis for New Therapeutic Approaches (METSY), Paris-Saclay University, 114 rue Edouard Vaillant, 94805 Villejuif, France; 3 Department of Pharmacology, Gustave Roussy, Paris-Saclay University, 114 rue Edouard Vaillant, 94805 Villejuif, France

**Keywords:** Bone, Electrochemotherapy, Spinal cord injuries, Spinal neoplasms, Swine

## Abstract

**Background:**

Electrochemotherapy (ECT) of vertebral metastasis is a new treatment option for metastasis that is not accessible to thermal ablation or radiotherapy. A numerical feasibility study has investigated the transpedicular approach for electrode insertion. We conducted a preclinical study to assess its safety.

**Methods:**

Histologic examination of the spinal cord was performed in 12 consecutive pigs treated with ECT at three consecutive levels (T11, T12, and L1) to evaluate any cellular or vascular damage. Pigs of group A (*n* = 6) had an intraoperative neuromonitoring immediately for 1 h after ECT and then were euthanized. Pain and clinical symptoms were daily evaluated for group B (*n* = 3) and group C (*n* = 3) until day-3 and day-30, respectively.

**Results:**

At gross pathology, no apoptosis, no vascular/thrombosis or hemorrhagic focus was observed in any pig. Motor-evoked potential responses of the lower limbs were transiently lost in response in 5 of the 6 pigs, but complete recovery always occurred within 30 min. Clinical examination (groups B and C) revealed no symptoms during the follow-up. Pigs were all able to walk normally, without weakness or paralysis of the lower extremities. No urinary/fecal retention or incontinence was observed, nor any sign of pain.

**Conclusion:**

Our results confirm that the insertion of electrodes through the pedicles is safe for the ECT of vertebral metastases. Further studies are needed to evaluate the safety profile of ECT of vertebral metastases invading the cortical and epidural fat, which represents a privileged pathway for the electric field between the electrodes.

**Relevance statement:**

Electrochemotherapy of vertebral metastases should be performed using a transpedicular approach for the insertion of electrodes, without definitive sequelae at the spinal cord level.

**Key Points:**

- Electrochemotherapy is a new treatment for vertebral metastases not accessible to radiotherapy, but it could result in spinal cord injury related to electrical trauma.
- In a swine model, the transpedicular approach has demonstrated no definitive sequelae at intraoperative neuromonitoring and during clinical follow-up.
- Electrochemotherapy should be performed using a transpedicular approach to avoid spinal cord damage.

**Graphical Abstract:**

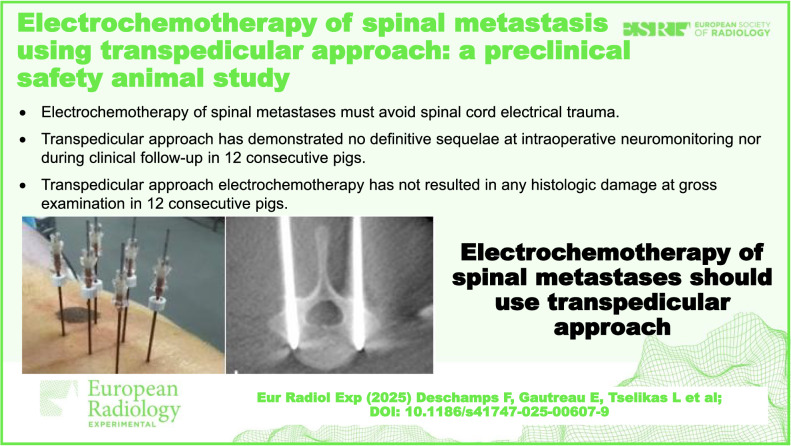

## Background

Electrochemotherapy (ECT) is a minimally invasive therapy that can be used for the locoregional treatment of bone metastasis [1]. ECT refers to the concomitant application of reversible electroporation and intravenous administration of bleomycin, with ECT enhancing bleomycin uptake into the cells. Electroporation is achieved through the percutaneous insertion of needle-like electrodes in or around the tumor, and subsequently, high-intensity electric pulses are delivered between pairs of needles to cause cellular membrane damage. The degree of damage depends upon the electric pulse field amplitude and the number of applied pulses.

Exceeding a threshold of 400 V/cm can cause temporary damage, termed reversible electroporation. Exceeding a threshold of 800 to 1,000 V/cm can result in permanent loss of cell homeostasis and cell death, termed irreversible electroporation [2]. Hence, while irreversible electroporation may not be ideal for vertebral metastases due to concerns for permanent spinal cord injury, the temporary damage caused by reversible electroporation may provide a novel and unique treatment opportunity.

ECT offers theoretical advantages for the treatment of vertebral metastases compared to other locoregional treatments, such as thermal-ablative techniques. First, bleomycin’s mechanism of action induces mitotic death, which is theoretically safe, or at least less toxic, for nonmitotic cells such as neurons [3, 4]. Second, ECT does not cause necrosis of the bone and does not prevent new bone formation [4, 5]. Finally, a recent study reports the evidence that ECT can rescue radiotherapy-resistant metastatic epidural spinal cord compression [6].

However, spinal cord damage can occur after ECT of metastatic epidural spinal cord compression related to the inevitable technological reality that hyperthermia and irreversible electroporation occur in the immediate vicinity of the electrodes [7]. To decrease this risk, the transpedicular approach has been investigated in a numerical feasibility study [8] and considered theoretically safe if the majority of the tumor volume is contained within the vertebral body.

We designed a preclinical study on pigs to investigate the potential effects of ECT on spinal cord integrity using this transpedicular approach, using an animal (swine) model.

## Methods

To investigate the potential toxicity of ECT on spinal cord integrity using the transpedicular approach, we performed ECT on 12 consecutive pigs (mean weight 40.3+/-3 kg, range: 35–45). The experimental design was approved by the Ethical Committee for Animal Experiments, Paris, France (APAFIS #33903-2021111517311128 v3).

All procedures were performed in the prone position under general anesthesia. Pigs were premedicated with an intramuscular injection of 3.5 mg/kg of tiletamine and 3.5 mg/kg of zolazepam (Zoletil®, Vibrac) anesthesia was induced with intravenous 3 mg/kg of propofol (Propovet®, Zoetis), and maintained with continuous 3% sevoflurane inhalation. Neuromuscular blockade was obtained using a bolus injection of 2 mg/kg of atracurium (Tracrium®, Aspen). Pain during the procedure and immediate postoperative pain were prevented using an intramuscular injection of 0.04 mg/kg of Buprenorphine (Buprecare®, Axience).

Percutaneous insertion of 6 electrodes was performed in an angio suite equipped with a cone-beam computed tomography (Discovery IGS 7, GE Healthcare) by an interventional radiologist with 15 years of experience in bone procedures. Electrodes were 14-gauge in diameter and with an active part of 20-mm in length (18/20T16 IGEA). They were inserted using a parallel bipedicular approach at three consecutive levels: T11, T12, and L1. Electrodes inserted through the left pedicles were numbered 1, 3, and 5 at T11, T12 and L1 levels, respectively. Electrodes inserted through the right pedicles were numbered 2, 4, and 6 at T11, T12 and L1 levels, respectively.

**Fig. 1:**
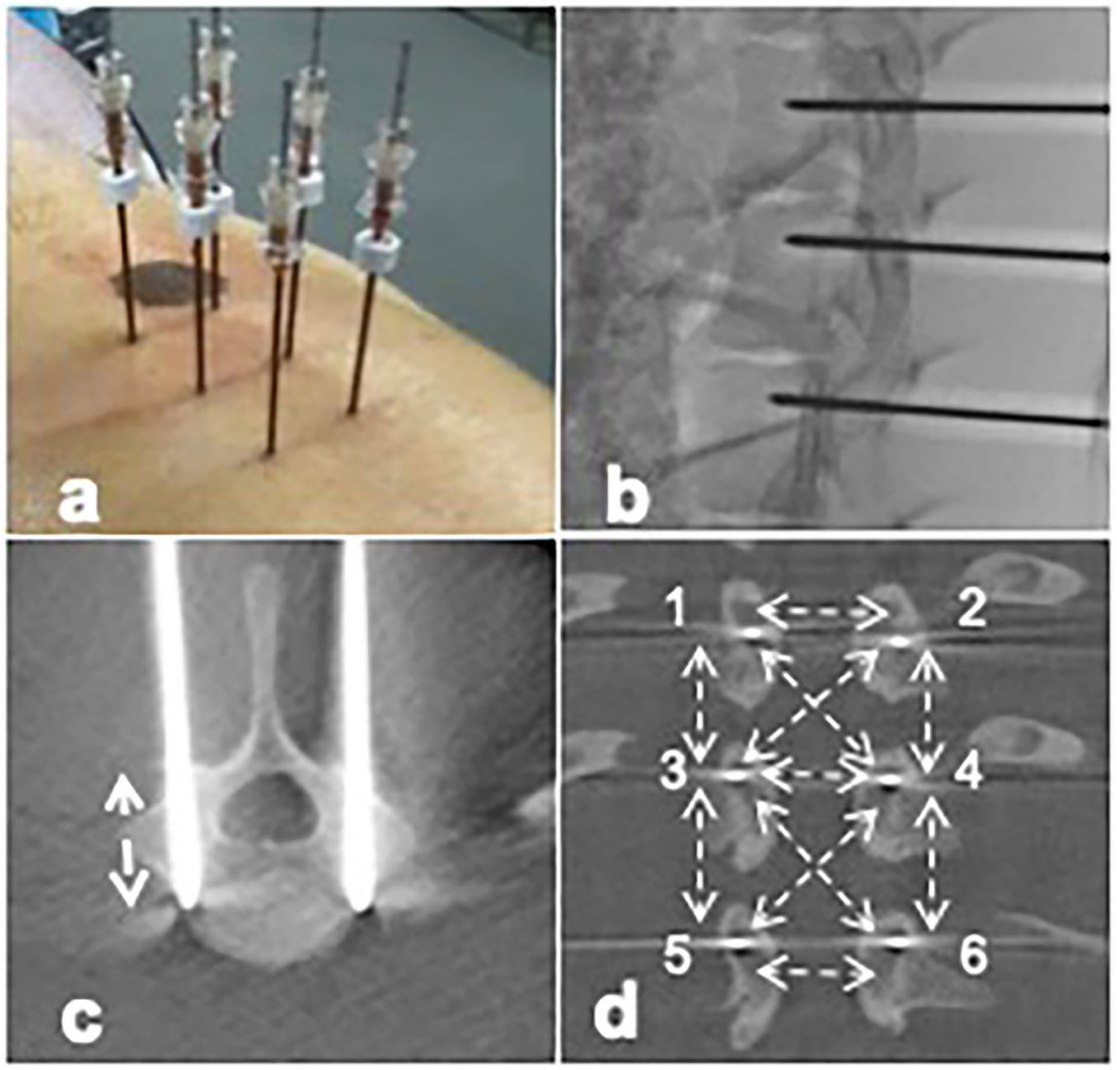
Percutaneous insertion of 6 electrodes (**a**) was performed under fluoroscopic guidance (**b**) using a bilateral transpedicular approach at three consecutive levels (T11, T12, and L1). The tip of each electrode was advanced in order to expose the active part (20 mm) at the level of the central canal and the spinal cord (**c**). Distances between electrodes were measured with cone-beam computed tomography (**d**) in order to deliver electroporation impacts using a voltage-to-distance ratio of 1,000 V/cm between all electrodes at adjacent levels, with a maximum voltage of 3,000 V

The tip of each electrode was advanced in order to expose the active part at the level of the central canal and the spinal cord. Distances between electrodes were measured with cone beam computed tomography acquisition (Fig. 1). All electrodes were connected to a pulse generator (Cliniporator Vitae, IGEA). Eight minutes after a bolus intravenous injection of 15 mg of bleomycin (Bleomycin, Sanofi®), electroporation was performed by means of two series of four electric pulses of opposite polarity of 100 μs individual duration and at 1 kHz pulse repetition frequency.

In order to evaluate potential toxicities of ECT, a worst-case scenario was used to mimic the treatment of a spinal metastasis located at T12: electroporation pulses were delivered using a voltage-to-distance ratio of 1,000 V/cm with activation of all pairs of electrodes between adjacent vertebrae, with a maximum voltage of 3,000 V.

The pigs were divided into three groups. Group A included 6 pigs and was used to investigate the acute effect of ECT on spinal cord integrity. For this purpose, we performed intraoperative neuromonitoring. Pigs in group A were euthanized at the end of the procedure (1 h after the electric pulses delivery) using an intravenous overdose of pentobarbital (Exagon®, Axience). The groups B and C included three pigs each and were used to investigate the subacute effects of ECT on spinal cord integrity. For this purpose, we daily evaluated their pain scale during the follow-up using the UNESP-Botucatu pig composite acute pain scale [6]. This scale is composed of six items, each with four subitems, providing a score ranging from 0 (normal) to 18 points (worst pain). Items are: posture, interaction and interest in the surroundings, activity, appetite, attention to the affected area and miscellaneous behaviors. Pigs in groups B and C were awakened at the end of the procedure and were euthanized at day-3 and day-30, respectively. For euthanasia, pigs in groups B and C were anesthetized using Zoletil® and received an intravenous overdose of pentobarbital (Exagon®).

Intraoperative neuromonitoring is used in many neurosurgical interventions to ensure that nerve functionality is preserved. By electrically stimulating the neural tissue, action potentials are elicited, and responses can be recorded from innervated muscles (for efferent fibers) and from cortical areas (for afferent fibers). By monitoring amplitudes and latencies of these responses, changes in signal conduction along the nerve tracks can be detected. Unchanged signal conduction ensures that nerve tissue is unaffected by the surgery, while particularly abrupt decreases of response amplitudes or increases of stimulation thresholds are hints that nerve preservation is at risk [9]. In this trial, intraoperative neuromonitoring (Isis Xpress, Inomed®) was used to observe whether the ECT has an immediate effect on nerve conduction and how nerve conduction evolves within the first hour after the application of the therapy.

**Fig. 2:**
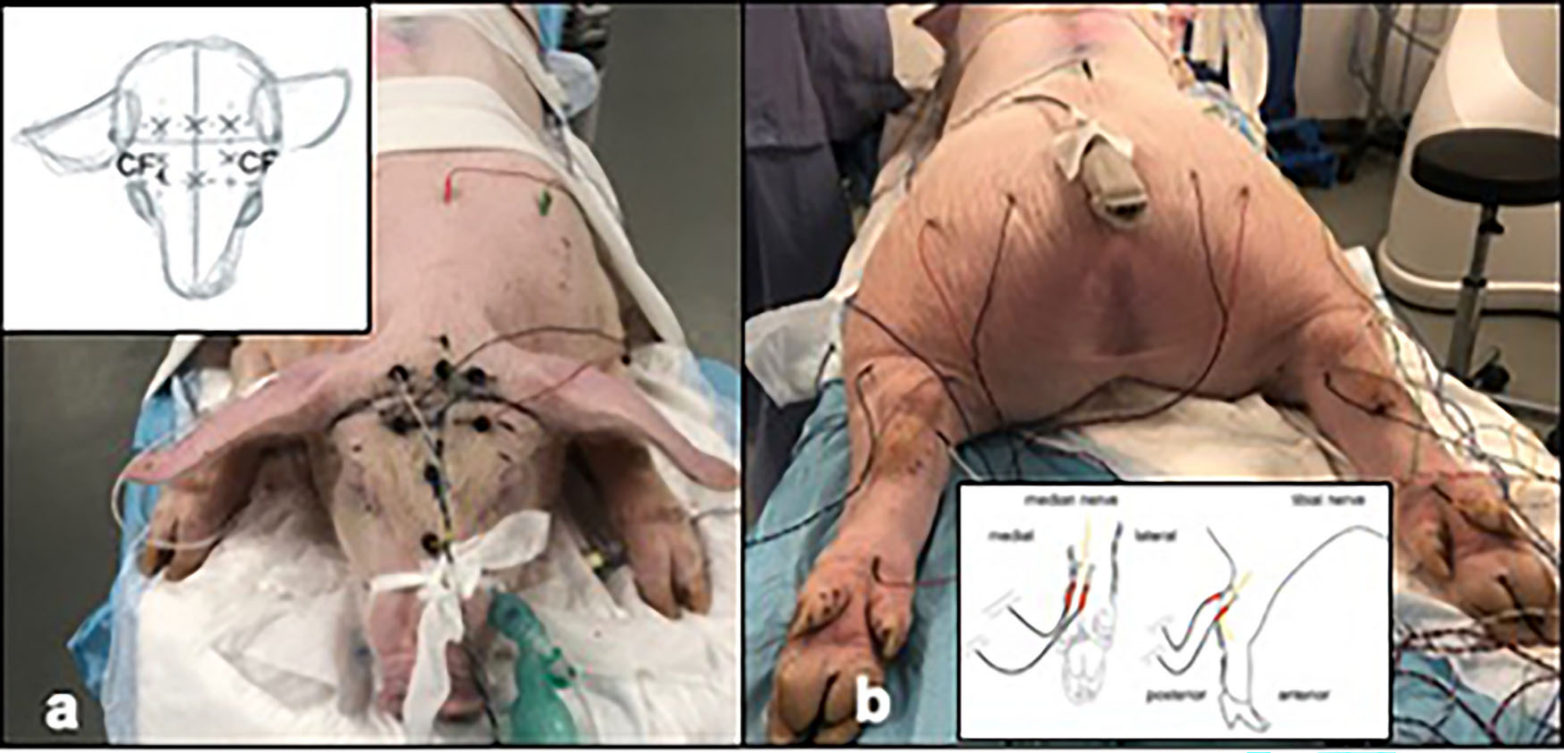
All procedures were performed in the prone position under general anesthesia. Intraoperative neuromonitoring was performed using electrodes on the skull (**a**) and on the upper and lower limbs (**b**)

In order to interpret the signals correctly, the pigs in group A were anaesthetized without neuromuscular blockade. The positioning of the electrodes for intraoperative neuromonitoring is illustrated in Fig. 2. After electrode placement, signal quality was determined. Electrodes were replaced, if necessary, to obtain the best possible signal quality. Before electroporation, baseline somatosensory-evoked potentials (SEP) and motor-evoked potentials (MEP) were recorded. After electroporation, SEP and MEP were measured every 2 min for the first 10 min and then every 10 min until 1 h. Signals were compared to the baseline signals to evaluate any acute change in nerve conduction after ECT.

**Fig. 3:**
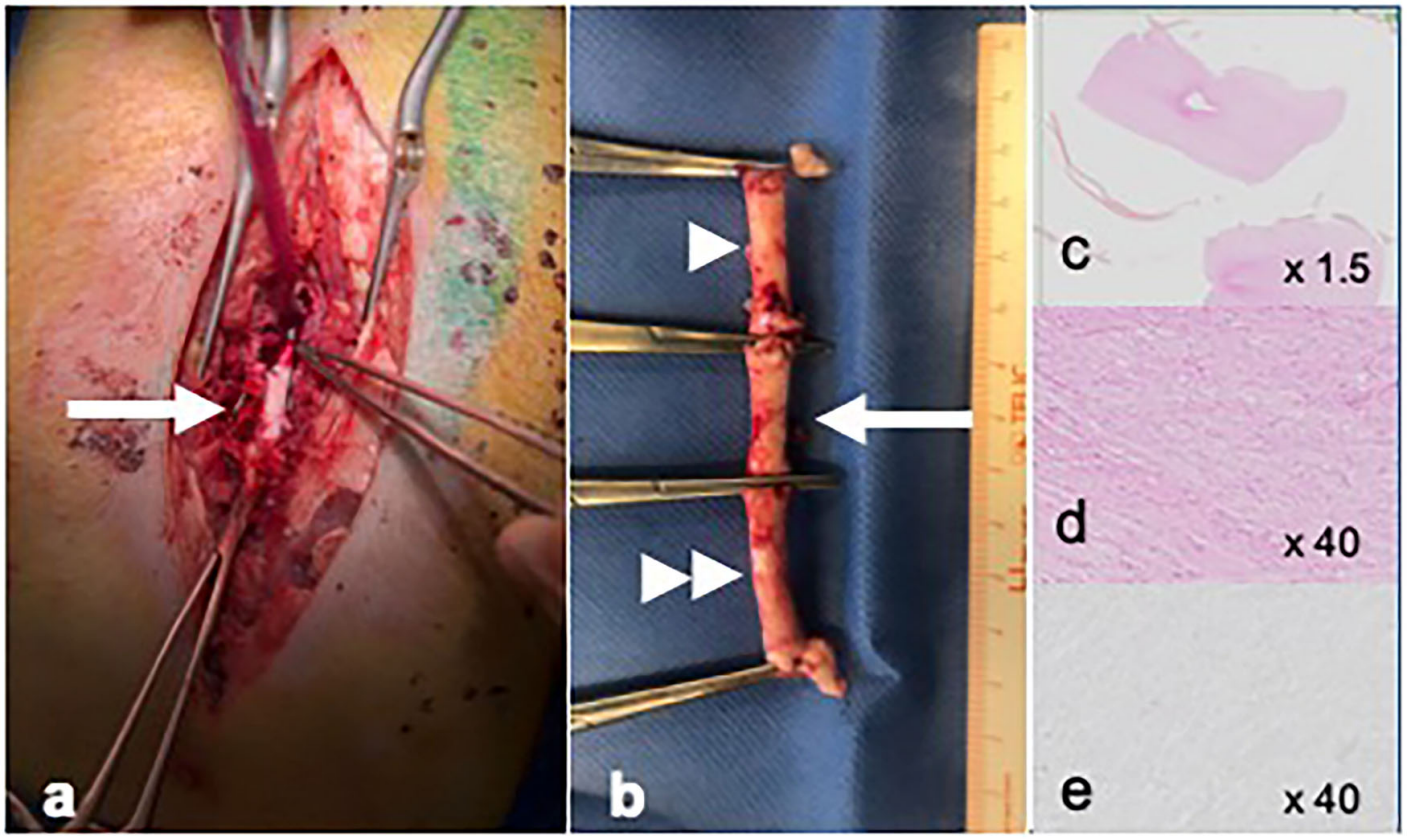
Surgical specimens from the spinal cord were collected for histologic examination at the time of sacrifice (**a**). One sample located at the treatment site (white arrow), one fragment upstream (single white arrowhead) and one downstream (double white arrowhead) (**b**). Gross pathology of the spinal cord at the same level of the treatment area from an animal sacrificed 30 days after the electrochemotherapy: at ordinary hematoxylin and eosin staining (**c**, **d**) and with TUNEL technique (**e**). TUNEL, Terminal deoxynucleotidyl transferase deoxyuridine triphosphate nick-end labeling

Histologic examination of the spinal cord was also performed in all pigs to evaluate any cellular or vascular damage 1 h after ECT (group A, *n* = 6), 3 days after ECT (group B, *n* = 3) and 30 days after ECT (group C, *n* = 3). Surgical specimens from spinal cord were collected from each animal, at the time of sacrifice, according to the following procedure: one sample located at the treatment site (at the level of T12 vertebra), one fragment upstream and one downstream (Fig. 3). Tissue samples were successively formalin-fixed and paraffin-embedded in order to cut 4-µm thick sections. The slides were then hematoxylin and eosin-stained. A total of 18 slides were analyzed for each animal. Terminal deoxynucleotidyl transferase deoxyuridine triphosphate nick-end labeling (TUNEL) assay [10] was also systematically performed on all the tissue samples, as well as γ-H2AX immunostaining [11] to respectively assess apoptosis and early deoxyribonucleic damages. Slides were also extensively analyzed under the microscope to assess any tissue damage. Elementary marks of tissue injury that the pathologist looked for included necrosis, cell apoptosis, hemorrhagic suffusions, vascular injury and perivascular inflammatory infiltrates.

## Results

Procedures were completed according to the protocol in 12 pigs. No complications occurred during the procedures. All pairs of electrodes were successfully placed and activated, and current was delivered across the spinal cord at a voltage of 1,000 V/cm. Table 1 reports the voltage delivered between the pairs of electrodes. Briefly, seven pairs of electrodes delivered electrical current across the central canal with 2,590 ± 510 V (mean ± standard deviation) each and four pairs of electrodes delivered electrical current laterally to the central canal with an average of 2,620 ± 20 V each.

**Table 1:** Mean distances and mean voltages between pairs of electrodes

Pairs of electrodes	Distance between electrodes (mm)	Voltage applied (V)
Across the central canal	31.7 ± 6.8 (16–35)	2,590 ± 510
1-2; 3-4; 5-6	20 ± 0.4 (16–28)	2,230 ± 40
1-4; 2-3; 3-6; 4-5	32.9 ± 0.9 (31–35)	3,000
Laterally to the central canal		
1-3; 2-4; 3-5; 4-6	26.8 ± 0.6 (25–27)	2,680 ± 60

Data are given as mean ± standard deviation (minimum−maximum range)

**Fig. 4:**
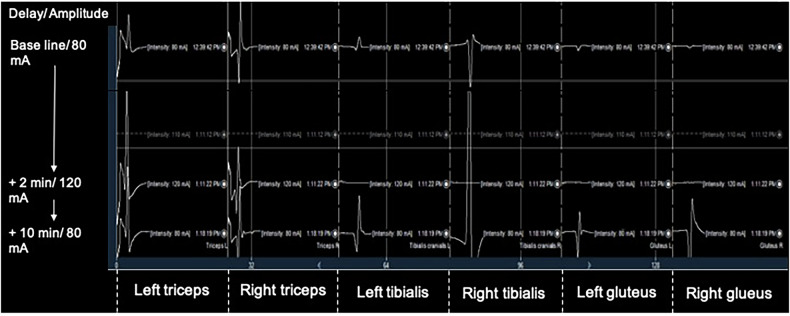
Motor-evoked potentials of the triceps (left/right) were recorded immediately after treatment, but an increased stimulation amplitude (120 mA instead of 80 mA) was necessary to detect them after the treatment. Conversely, the muscles from the hind limbs (tibialis cranialis and gluteus) displayed a lower amplitude as baseline, disappeared after treatment, even at recording amplitudes 1.5-times the baseline threshold, but then returned 10 min after treatment with a much higher intensity

Intraoperative neuromonitoring was performed in the first six pigs (group A). MEP responses of the hind limbs could be evoked in all six animals, with stimulation currents ranging from 80 to 140 mA. MEP responses of the upper limbs were never influenced by the treatment. MEP responses of the lower limbs were completely lost in response to the ECT treatment in five of six animals (Fig. 4) and remained unchanged in one animal. The time between treatment and complete recovery of the MEP amplitudes varied between 7 and 30 min (mean 13 min). SEP recordings were unstable, independent of ECT. SEP of the upper limbs could be obtained in all six animals and showed no change during the procedure in three cases; in the other three cases, only one had clear changes, time-related to the treatment (increase of latency). SEP of the lower limbs could be evoked in two of six animals and were lost when the ECT treatment was administered. In one case, the SEP responses took 20 min to recover (compared to 30 min for MEP recovery in the same animal). In the second animal in which SEP could be elicited from the lower limbs, the recordings did not clearly display modifications due to the treatment (recording was impossible to interpret since the animal was shaking until anesthesia was changed). However, at the end of the experiment, the evoked SEP was similar to the baseline.

Clinical examination was performed daily in the six pigs belonging to groups B and C. No symptoms appeared within hours of waking or during the follow-up. They were all able to walk normally immediately after the procedure, without weakness or paralysis of the lower extremities. No urinary retention, nor urinary and/or fecal incontinence was observed. No sign of pain in the back and/or legs was noticed. The UNESP-Botucatu pig composite acute pain scale [12] remained normal (0 out of 18) in all pigs every day during the follow-up.

Surgical specimens from the spinal cord were collected in all the animals at the time of sacrifice. At ordinary hematoxylin and eosin staining, native cell populations could be identified. The samples were mainly, but not exclusively, constituted by white matter. Glial cells appeared regularly shaped, with ovoid or round nuclei and without relevant cytological alterations. In the inner part of the spinal cord sample, these cells stand close to neuronal cells, which appeared larger and slightly hyperchromatic. No feature consistent with apoptosis could be identified upon morphological analysis. Dendrites and axons appeared normally intermingled and branched. No vascular change was observed, as well as no thrombosis or hemorrhagic focus. Both the TUNEL assay and γ-H2AX immunostaining were unable to reveal cell damage in any pig. No edema was identified. Histological examination showed no difference between the fragments of the spinal cord located at the treated level and the fragments located upstream and downstream. No difference was observed between pigs sacrificed at 1 h (group A), pigs sacrificed at 3 days (group B) or pigs sacrificed at 30 days (group C).

## Discussion

Electrocution can result in neurological consequences, especially after high-voltage injury. Most data come from observations of clinical consequences after electrical trauma and lightning injury [13, 14]. The hypothetical pathophysiological mechanisms are vascular damage, electroporation of cells, alteration of proteins and/or DNA, and oxidative stress leading to demyelination [15]. The use of electroporation enhances bleomycin uptake into the tumor cells and offers great hope to rescue radiotherapy-resistant spine metastases close to the spinal cord [3]. A recent retrospective study demonstrated that tumor responses were obtained in 77% of the patients at the 1-month MRI (46% of complete responses and 31% of partial responses). However, the rate of paraplegia in the study was 7.5%, underlining the need for further research to improve the safety profile of ECT.

Our study was designed to evaluate the potential toxicity of ECT for the treatment of spine metastasis without epidural impingement or spinal cord compression, corresponding to grade 0 on the Epidural Spinal Cord Compression scale (Table 2) [16]. The treatment of three consecutive vertebrae with electrodes inserted on each side of the spinal cord was considered the worst-case scenario for the risk of spinal cord damage, especially since a voltage of 1,000 V/cm was delivered between all pairs of electrodes of consecutive vertebrae. ECT is based on the achievement of “reversible” electroporation, which results in the transient electropermeabilization of the cells without impairment of their viability. Therefore, in ECT, irreversible electroporation of the tissues must be avoided or reduced as much as possible to prevent permanent changes in cell permeability, and thus cell death in the normal tissues, including nerve cells, and damage to the spinal cord in our case. In this study, we decided to inject bleomycin during the procedures even if there was no tumor in the vertebrae of the pigs. Indeed, our purpose was to evaluate the toxicity of ECT and not of the electric pulses only. Since electroporation could result in a neurological toxicity related to the enhancement of bleomycin uptake into the neurons, we decided to perform all steps of the procedure with systemic injection of bleomycin and electropermeabilization.

**Table 2:** Epidural spinal cord compression scale [8]

Grade 0	Vertebral bone involvement only without canal compromise
Grade 1	Minimal involvement of the epidural fat but without thecal sac displacement
Grade 2	Impingement and/or displacement of the thecal sac
Grade 3	Impingement of the spinal cord, but without significant distortion or displacement of the spinal cord
Grade 4	Spinal cord compression and displacement with cerebrospinal fluid still visible within the thecal sac

Our study has some limitations. First, the number of animals is limited, and the absence of long-term toxicity is based on three pigs only, without symptoms at 30 days. Follow-up was also limited (30 days), and some authors report that a latent period between electrical shock and neurologic complications might be observed because structural changes are not sufficient to cause immediate cell death, resulting in delayed cell death and thus delayed neurologic symptoms [12, 13]. Another limitation is the absence of bone tumors in the vertebrae of the pigs, allowing conclusions only for metastasis grade 0 on the epidural spinal cord compression scale, which does not affect the insulating cortical bone tissue or the fat layer.

Nevertheless, it is worth noting that no neurological toxicity was observed clinically or at the histological examinations. Pigs in groups B and C displayed no neurological deficit during the follow-up, and nervous tissue appeared well-preserved, without alterations at 3 and 30 days. These results suggest that the transpedicular approach results in an electric field that preserves the spinal cord from severe electrical damage, probably because the bone cortex of the pedicles and epidural fat have low conductivity properties, partly shielding the spinal cord. However, electricity is certainly passing through the spinal cord despite these insulating structures, since five of six pigs in group A completely lost MEP responses in lower limbs for several minutes after ECT. These neurological deficits revealed by intraoperative neuromonitoring were always transient and recovery of the initial MEP amplitudes occurred in all animals. We believe that this side effect was consistent with the depolarization/repolarization phenomenon that occurs at the cell membranes after electrical changes of their transmembrane potential. Thus, the voltage through the spinal cord was low enough to avoid serious electrical injury but high enough to have transient neurological effects detectable by intraoperative neuromonitoring.

It is worth noting that our protocol used electrodes with a diameter of 14 gauge and an active part of 20 mm in length, which was consistent with the dimensions of the pig vertebrae and with a containment of the electric current to the vertebral body. Based on our results, we assume that ECT delivered using a transpedicular approach and a field amplitude of 1,000 V/cm is quite safe for the treatment of vertebral metastasis when the bone cortical of the pedicles is intact and epidural fat surrounds the spinal cord, *i.e*., grade 0 on the Epidural Spinal Cord Compression−ESCC scale [16]. Greater caution should be taken every time that the electric field is not as confined as in the case of our study, for example, in the case of using electrodes with a longer active part or in the case of grade 1, 2, and 3 lesions on the Epidural Spinal Cord Compression scale. The cord will be less shielded because the tumor may invade the cortical and the epidural fat, representing a privileged pathway for the electric field between the two electrodes. Under these last configurations, different strategies can be foreseen, such as the use of a decreased voltage-to-distance ratio between the pairs of electrodes or different geometrical configurations of the electrodes, to avoid a high electric field through the central canal and to decrease the risk of definitive sequelae [17].

Our results confirm a numerical study [8] that demonstrated that the insertion of electrodes through the pedicles seems safe if the tumor volume is contained within the vertebral body. In that study, the authors stated that the transpedicular approach results in an electric field distribution that covers close to 100% of the spine tumor in three representative cases. However, the authors also stressed the fact that if the tumor has grown into the pedicle area or into the region of the vertebral arch, electroporation is not able to cover these regions of tumor volume with a sufficiently high electric field without risk of damage to the spinal cord tissue [8]. In those cases, an appropriate positioning of the electrodes and a reduction of the voltage-to-distance ratio may suffice to maintain the efficacy of the ECT and ensure the safety of the procedure. Further studies are required to investigate these parameters in terms of safety and efficacy.

## Data Availability

Data supporting the results are available from Frederic Deschamps at Frederic.deschamps@gustaveroussy.fr.

## Abbreviations

ECT: Electrochemotherapy
MEP: Motor-evoked potentials
SEP: Somatosensory-evoked potentials
TUNEL: Terminal deoxynucleotidyl transferase deoxyuridine triphosphate nick-end labeling
